# Efficacy of neural stem cell transplantation for the treatment of patients with spinal cord injury: A protocol of systematic review and meta-analysis

**DOI:** 10.1097/MD.0000000000020169

**Published:** 2020-05-08

**Authors:** Hua-yu Tang, Yu-zhi Li, Zhao-chen Tang, Lu-yao Wang, Tian-shu Wang, Fernando Araujo

**Affiliations:** a Second Ward of Orthopedics Department; b Department of Urology, First Affiliated Hospital of Jiamusi University; c School of Clinical Medicine, Jiamusi University, Jiamusi, China; d University of Bristol, Bristol, UK.

**Keywords:** efficacy, neural stem cell transplantation, safety, spinal cord injury

## Abstract

**Background:**

The aim of this study is to evaluate the efficacy of neural stem cell transplantation (NSCT) for the treatment of patients with spinal cord injury (SCI).

**Methods:**

All potential randomized controlled trials (RCTs) on NSCT in the treatment of patients with SCI will be searched from the following electronic databases: Cochrane Library, MEDILINE, EMBASE, Web of Science, Scopus, CBM, WANGFANG, and CNKI. We will search all electronic databases from their initiation to the January 31, 2020 in spite of language and publication date. Two contributors will independently select studies from all searched literatures, extract data from included trials, and evaluate study quality for all eligible RCTs using Cochrane risk of bias tool, respectively. Any confusion will be resolved by consulting contributor and a consensus will be reached. We will utilize RevMan 5.3 software to pool the data and to conduct the data analysis.

**Results:**

This study will summarize the most recent RCTs to investigate the efficacy and safety of NSCT in the treatment of patients with SCI.

**Conclusion:**

This study will provide evidence to assess the efficacy and safety of NSCT in the treatment of patients with SCI at evidence-based medicine level.

**Systematic review registration:**

PROSPERO CRD42020173792.

## 1 Introduction

Spinal cord injury (SCI) is a common disabling and devastating neurological disease that often causes long-term impairments in physical function and psychological status.^[1–4]^ It is reported that the prevalence of SCI was about 27.04 million cases, and the new cases was 0.93 million in 2016.^[5]^ It often manifests as the permanent loss of voluntary movement, sensation, and function below the site of the injury,^[6–9]^ which can dramatically reduce quality of life in patients with SCI.^[10–12]^

A variety of studies have reported that neural stem cell transplantation (NSCT) has been utilized for the treatment of SCI.^[13–30]^ However, no systematic review has assessed the efficacy and safety of NSCT for the treatment of patients with SCI. Therefore, this study will appraise the efficacy and safety of NSCT for the management of SCI.

## 2 Methods

### 2.1 Study registration

This study protocol has been funded and registered on PROSPERO CRD42020173792. We report this study in accordance with the Cochrane Handbook for Systematic Reviews of Interventions and the Preferred Reporting Items for Systematic Reviews and Meta-Analysis Protocol statement guidelines.^[31]^

### 2.2 Dissemination and ethics

This study is expected to be disseminated at a peer-reviewed journal or relevant conference meeting. Since this study will not obtain privacy data, thus no ethical approval is needed.

### 2.3 Inclusion criteria for study selection

#### 2.3.1 Types of studies

All randomized controlled trials (RCTs) that applying NSCT as the treatment for patients with SCI will be brought into this study. We will not apply any limitations to the language and publication date.

#### 2.3.2 Types of participants

Any adult patients (18 years old or over) diagnosed with SCI will be included in this study regardless their ethnicity, sex, age, and the length and severity of disease.

#### 2.3.3 Types of interventions

The patients in the treatment group received NSCT as their treatment.

The patients in the control group underwent any therapies for the treatment, but not any forms of NSCT.

#### 2.3.4 Type of outcome measurements

Primary outcome are spasticity (as measured by any relevant validated scales, such as Modified Ashworth Scale), and walking ability (as assessed by any related validated tools, such as 10 m-Walk Test).

Secondary outcomes are pain intensity (as investigated by any validated pain scores, such as Numeric Rating Scale), health-related quality of life (as examined any associated validated questionnaires, such as 36-Item Short Form Survey), duration of stay at hospital (days), mortality rate, and incidence of any expected or unexpected adverse event.

### 2.4 Search methods for the identification of studies

#### 2.4.1 Electronic database searches

A systematic and comprehensive search will be carried out in the following electronic databases from their initiation to the January 31, 2020 in spite of language and publication date: Cochrane Library, MEDILINE, EMBASE, Web of Science, Scopus, CBM, WANGFANG, and CNKI. All potential randomized controlled trials (RCTs) on investigating the efficacy and safety of NSCT in the treatment of patients with SCI will be considered for inclusion. Detailed search strategy of Cochrane Library will be exerted (Table 1). We will also modify similar search strategies for other electronic databases.

**Table 1:**
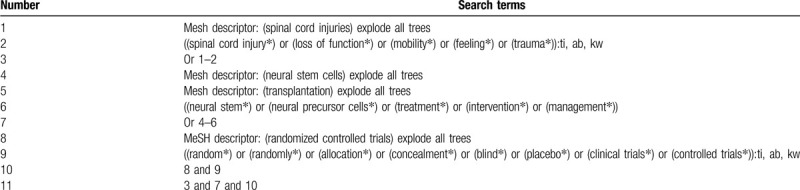
Search strategy for Cochrane Library database.

#### 2.4.2 Search for other resources

To avoid missing potential trials, we will also retrieve conference papers, dissertations, ongoing studies, and reference list of all related reviews.

### 2.5 Data collection and analysis

#### 2.5.1 Study selection

**Figure 1:**
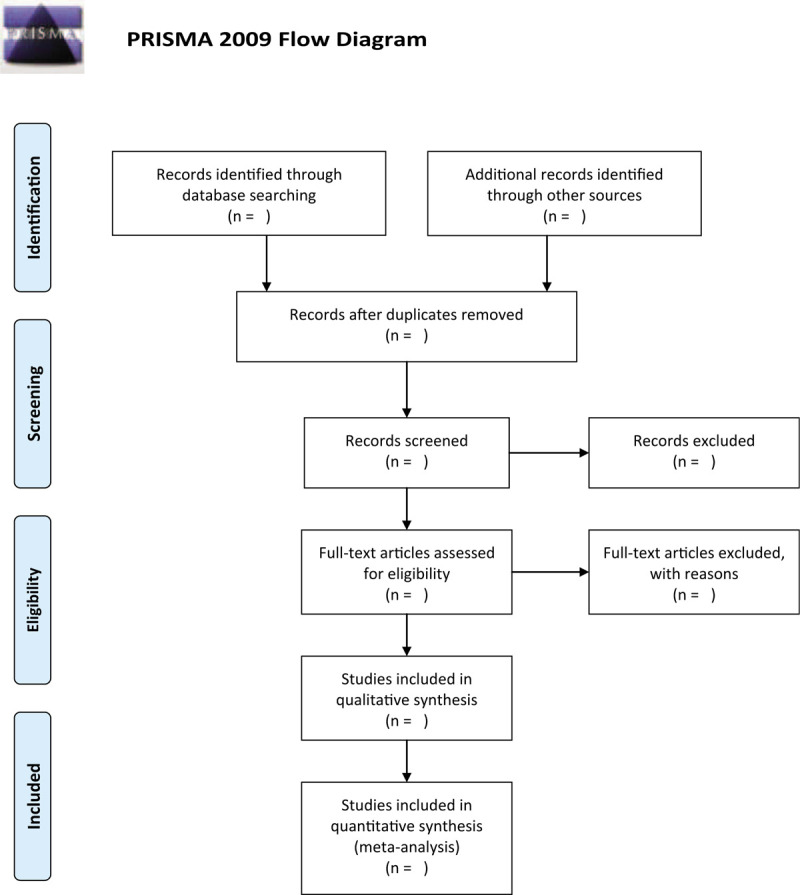
Flow diagram of study selection.

Selection of studies will be independently conducted by 2 contributors and will be cross-checked between them. Any different views between them will be solved by discussion with the help of another contributor. All collected documents will be imported into EndNote X9 and all duplicated literatures will be removed. Then, titles and abstracts of all records will be scanned to rule out obvious nonconformities. After that, we will obtain full-texts of remaining studies and will carefully examine them according to the eligibility criteria. The entire filtering procedure will be presented in a flowchart (Fig. 1).

#### 2.5.2 Data collection and management

After study selection, all eligible trials are included, and essential data will be extracted from all of them by 2 contributors using a predetermined sheet of data collection. Any inconsistencies will be discussed and negotiated with another contributor. The extracted data includes title, first author, publication year, sample size, patient characteristics (such as race, sex, age, eligibility criteria, and severity and duration of SCI), trial setting, trial methods (such as details of randomization and blind), specifics of treatment and comparators (such as deliver types, dosage, and frequency), outcomes, safety, and other relevant data. If we identify any missing or incomplete data, we will contact original corresponding authors to request them.

#### 2.5.3 Study quality assessment

Two contributors will independently assess study quality using Cochrane Handbook for Systematic Reviews of Interventions tool. This tool appraises study quality of each trial through 7 sectors, and each item will be further assessed as low, unclear, or high risk of bias. Any potential divergences will be discussed with the help of another contributor, and a final decision will be reached.

#### 2.5.4 Measurement of treatment effect

To appraise the treatment effect for continuous data, mean difference or standardized mean difference and 95% confidence intervals (CIs) will be used. For enumeration data, risk ratio and 95% CIs will be calculated.

#### 2.5.5 Assessment of heterogeneity

The statistical heterogeneity will be examined by *I*^2^ test. When *I*^2^ ≤ 50%, heterogeneity is acceptable, and a fixed-effects model will be adopted, while when *I*^2^ > 50%, heterogeneity is obvious, and a random-effects model will be employed.

#### 2.5.6 Data synthesis

We will employ RevMan 5.3, (Cochrane Community, London, UK) software to synthesize and analyze the data, and to perform a meta-analysis if possible. If acceptable heterogeneity is examined among included trials, we will conduct a meta-analysis in accordance with the few variations in study and patient characteristics, and few differences in treatments, controls, and outcomes. If considerable heterogeneity is identified, we will carry out subgroup analysis and sensitivity analysis to find out any possible sources of obvious heterogeneity. If it is impossible to undertake a meta-analysis, we will report study results as a narrative summary.

#### 2.5.7 Publication bias

When the eligible studies are sufficient (over 10 RCTs), the reported bias will be visualized by funnel plot and Egger regression test.^[32]^

#### 2.5.8 Subgroup analysis

We will observe the source of considerable heterogeneity by subgroup analysis based on variations in study and patient characteristics, study quality, different interventions, comparators, and outcomes.

#### 2.5.9 Sensitivity analysis

We will perform sensitivity analysis to test the robustness and satiability of conclusions by removing low quality trials, and trials with small sample size.

## 3 Discussion

Clinical studies have shown that NSCT can be used for the management of SCI.^[13–30]^ Although related research-results suggest that NSCT in the treatment of SCI can effectively alleviate the clinical symptoms, and can also help improving their quality of life, there are still inconsistent conclusions of this issue at evidenced-based medicine level. The results of this study may provide systematical and comprehensive evidence of NSCT in the treatment of patients with SCI.

## Author contributions

**Conceptualization:** Hua-yu Tang, Yu-zhi Li, Zhao-chen Tang, Lu-yao Wang, Tian-shu Wang.

**Data curation:** Yu-zhi Li, Zhao-chen Tang, Lu-yao Wang.

**Formal analysis:** Yu-zhi Li, Zhao-chen Tang, Lu-yao Wang, Tian-shu Wang.

**Investigation:** Hua-yu Tang, Tian-shu Wang.

**Methodology:** Yu-zhi Li.

**Project administration:** Hua-yu Tang, Tian-shu Wang.

**Resources:** Hua-yu Tang, Yu-zhi Li, Zhao-chen Tang, Lu-yao Wang.

**Software:** Hua-yu Tang, Yu-zhi Li, Zhao-chen Tang, Lu-yao Wang.

**Supervision:** Hua-yu Tang, Tian-shu Wang.

**Validation:** Hua-yu Tang, Yu-zhi Li, Zhao-chen Tang, Lu-yao Wang, Tian-shu Wang, Fernando Araujo.

**Visualization:** Yu-zhi Li, Lu-yao Wang, Tian-shu Wang, Fernando Araujo.

**Writing – original draft:** Hua-yu Tang, Yu-zhi Li, Zhao-chen Tang, Lu-yao Wang, Tian-shu Wang, Fernando Araujo.

**Writing – review & editing:** Hua-yu Tang, Yu-zhi Li, Zhao-chen Tang, Lu-yao Wang, Tian-shu Wang, Fernando Araujo.
